# Fall Detection and Prevention Technologies for Older Adults: A Scoping Review

**DOI:** 10.1093/geroni/igaf122.2627

**Published:** 2025-12-31

**Authors:** Yong Kyung Choi, Ryan Osal, Armari Long, Haomin Hu, Steven Handler

**Affiliations:** University of Pittsburgh, Pittsburgh, Pennsylvania, United States; University of Pittsburgh, Pittsburgh, Pennsylvania, United States; University of Pittsburgh, Pittsburgh, Pennsylvania, United States; University of Pittsburgh, Pittsburgh, Pennsylvania, United States; University of Pittsburgh, Pittsburgh, Pennsylvania, United States

## Abstract

One in three older adults experience one or more falls each year. Such falls can have severe consequences, including injuries or death, significantly impacting older adults’ quality of life and independence. The economic burden is also substantial, with fall-related healthcare costs exceeding $31 billion. In response, digital and smart home safety technologies offer promising avenues for enabling timely interventions and enhancing fall detection and prevention. While prior research has developed and examined various fall-related technologies, rapid technological advancements, especially with the emergence of Artificial Intelligence (AI), necessitate exploring new and improved solutions. This scoping review examines recently published studies on digital tools designed for fall prevention and detection among older adults. A scoping review was conducted following PRISMA-ScR guidelines, identifying studies from PubMed, CINAHL, Medline (EBSCO), and Web of Science published between 2019 and 2024. This timeframe was selected to capture the most recent technological advancements and their applications in real-world settings. Studies were included if they evaluated digital tools specifically designed for fall prevention, detection, or risk assessment among older adults. From this review, 18 studies met the inclusion criteria, categorizing technologies into wearable sensors, motion capture and gait analysis tools, gamified prevention programs, robotics, smart home monitoring systems, and mobile applications. These digital tools support real-time fall risk assessment, provide personalized interventions, and integrate with health monitoring systems. However, challenges persist, including sensor synchronization issues, reliance on controlled settings, privacy concerns, and limited validation in diverse populations. Despite promising advancements, current research remains limited in scope and generalizability.

